# Screening the United States Blood Supply for West Nile Virus: A Question of Blood, Dollars, and Sense

**DOI:** 10.1371/journal.pmed.0030099

**Published:** 2006-01-24

**Authors:** Bruce Y Lee, Brad J Biggerstaff

## Abstract

Lee and Biggerstaff discuss two new studies on the cost effectiveness of different strategies for screening blood for West Nile virus.

Now that the media frenzy over West Nile virus (WNV) has subsided, and pictures of dead birds and insect repellant cans no longer permeate the nightly news, what shall we do about screening the United States blood supply for the virus? Shortly after the spread of WNV in North America and the revelation that blood transfusions may transmit the virus [ 1], US officials established regulations requiring blood banks to screen all their donated blood for WNV using nucleic acid amplification tests. Although these requirements have been in place since June 2003, there has been debate over if and how to continue the screening [ 2, 3]. The threat of disease certainly still exists, but with limited resources and other potential hazards to blood supply safety, there has been a real need for good, objective economic studies to determine what type and what degree of screening should be performed.

## Two New Cost-Effectiveness Studies

Two recent cost-effectiveness studies address this question, one by Korves and colleagues [ 4] published in *PLoS Medicine* (summarized in Box 1) and another by Custer and colleagues [ 5] published in a recent issue of *Annals of Internal Medicine* (summarized in Box 2). Discussing mass infectious disease screening measures without considering economic consequences is like eating at a smorgasbord without considering calories and fat. While in the short term, “indulging” in a certain screening measure may be necessary to avert a disease outbreak, in the long term, public health officials must decide if it is worth continuing to invest precious resources that may be better utilized in other areas.

Because study assumptions and methodological approaches can vary, a series of studies may be needed to move toward an appropriate policy. Both Korves's study and Custer's study arrive at similar general conclusions: the cost-effectiveness of individual donor screening depends on WNV prevalence, and targeted donor screening appears to be more cost-effective than mass donor screening. The two studies differ, however, in some of the questions that they address and in the answers that they provide.

### Who gets the blood may be as important as who gives the blood

While Custer's study focused on the blood donors, Korves's study also evaluated strategies that considered the recipient's immune status, finding that even when the prevalence of the virus is low, screening blood supplies destined for immunocompromised recipients may be cost-saving. The dangers and consequences of even a few immunocompromised patients contracting WNV by transfusion appear to significantly outweigh the low incidence of transmission.

### The question may be not only whether to pool but how much to pool

While both studies compared individual donated blood sample testing with minipool testing, Korves's study took the extra step of looking at minipools of 16 samples and minipools of six samples separately. In the minipool testing method, a number of individual samples are combined in a pool, and the pool of samples is tested. When the pool tests negative, one assumes that each individual sample that contributed to the pool is negative. When the pool tests positive, one tests the individual donations to find the positive(s). The advantage of the minipool method is that it reduces the number of tests that have to be run, presumably reducing costs and saving time. The major drawback of this “pooling” method is that testing may not be able to detect low levels of virus that are further diluted when blood samples are combined. Therefore, pooling six samples together is not the same as pooling 16 samples; for a given number of donations, pools of size 16 require fewer tests, but each test may be less sensitive because of dilution. Although Korves's study did not find significant differences in the cost-effectiveness of these two types of pooling, the balances in efficiencies and costs between virus detection assays and the mechanics of testing may, in the future, favor larger pools.

### Consider all of the steps involved in a screening operation

In determining the costs of screening, Korves's study captured aspects of the testing procedure—such as discarding false positives, notifying donors, and retrieving test results—which were overlooked by Custer's study. Screening can be a complicated operation and seemingly minor steps can inflate overall costs (especially when disproportionate time and labor are involved), or they can be bottlenecks. A cost-effectiveness study may suggest such targets for cost reduction.

### Seasonal screening is potentially very different from year-round screening

Korves's study assumed that the cost per sample screened would remain relatively constant between seasonal and year-round approaches. In reality, in seasonal screening, there are start-up costs at the beginning of the screening season (e.g., appropriate reagents have to be produced and put in place) and stoppage costs at the end of the screening season (e.g., the reagents have to be removed from the production and screening lines and disassembled), some of which were accounted for in Custer's study. Additionally, in seasonal screening, personnel and operations may not be as efficient as in year-round screening (e.g., it may take a while for things to get up to speed, and it may be tougher to find seasonal employees than year-round employees).

### When estimating the impact of disease, do not overlook potential productivity losses

In tabulating the cost of disease, Custer and colleagues included work productivity losses not considered by Korves and colleagues. Productivity losses from patients missing work or no longer able to work because of disability and death are always an important component of disease costs [ 6, 7]. This is especially true with a viral illness such as WNV, where the acute illness is often overshadowed by malaise and fatigue that may impair a person's ability to work.

## Conclusion

In the end, there are few perfect economic studies, and one should neither require nor expect perfection. Instead, the measure of an economic study is in not only the answers it provides but also the questions it raises. In this way, both Korves's article and Custer's article succeed. In addition to providing some answers (e.g., prevalence is related to cost-effectiveness, targeted screening may be more cost-effective, and blood designated for the immunocompromised should be screened), they raised different important questions (e.g., what are the true costs of seasonal and year-round screening? and what are the full economic effects of WNV?) and offered some essential direction for additional lines of inquiry. These studies indicate that the optimal cost-effectiveness strategy for WNV screening indeed depends on the situation, and public health officials can use their results in broader evaluations of WNV screening strategies.

**Box 1. Korves and Colleagues' 2006 Study**

Korves and colleagues constructed a Markov model simulating patients receiving blood transfusions under nine different screening strategies: (1) only administering a donor questionnaire, (2) year-round testing of 16-sample minipools (for a definition of minipool testing, see section entitled The Question May Be Not Only Whether to Pool, but How Much to Pool), (3) seasonal (i.e., May through November) testing of 16-sample minipools, (4) year-round testing of six-sample minipools, (5) seasonal testing of six-sample minipools, (6) year-round individual donor testing, (7) seasonal individual donor testing, (8) year-round individual testing of donations designated for immunocompromised recipients, and (9) seasonal individual testing of donations designated for immunocompromised recipients. They assumed that test-kit cost per sample would be US$3 for individual testing, US$0.50 for six-sample minipool testing, and US$0.19 for 16-sample minipool testing; determined a US$19 laboratory technician hourly rate; added additional testing costs such as the costs of discarded samples (US$90) and donor notification (US$500); and abstracted disease costs from the literature. They found that in high WNV prevalence areas, seasonal individual donation screening of blood designated for immunocompromised recipients would be most cost-effective and, in fact, cost saving, whereas in areas with low prevalence, using a donor questionnaire alone would be most cost-effective.

**Box 2. Custer and Colleagues' 2005 Study**

Custer and colleagues also constructed a Markov cohort model simulating patients receiving transfusions under seven different blood screening strategies: (1) no screening, (2) minipool testing throughout the US for half of the year, (3) minipool testing throughout the US over the entire year, (4) individual donor testing for one-third of the year in the quarter of the US with the highest prevalence of WNV, and minipool testing for the rest of the country and for the remaining two-thirds of the year, (5) individual donor testing for the entire year in the quarter of the US with the highest prevalence of WNV, and minipool testing for the rest of the country, (6) individual donor testing for one-third of the year, with minipool testing for the remainder of the year, and (7) national individual donor testing over the entire year. Using vendor reagent prices, they assumed that each minipool test would cost US$7 plus US$3 for labor and other related costs, each individual donation test would cost US$14 plus US$5 for labor and other related costs, and partial-year minipool testing and transitioning between minipool and individual donation testing would result in some additional laboratory preparation costs. Disease costs came from an economic study of the 2002 Louisiana WNV outbreak [ 8] and published productivity loss tables [ 9]. The most cost-effective strategy was annual, national minipool testing (US$483,000 per quality-adjusted life-year saved), and sensitivity analyses showed that the cost-effectiveness depended most heavily on WNV virus prevalence and testing costs.

## Abbreviation

WNV: West Nile virus
